# Role of Surface Preparation in Corrosion Resistance Due to Silane Coatings on a Magnesium Alloy

**DOI:** 10.3390/molecules26216663

**Published:** 2021-11-03

**Authors:** Abhishek Saxena, R. K. Singh Raman

**Affiliations:** 1 Department of Chemical and Biological Engineering, Monash University, Clayton, VIC 3800, Australia; 2abhisheksaxena@gmail.com; 2 Department of Mechanical and Aerospace Engineering, Monash University, Clayton, VIC 3800, Australia

**Keywords:** silane coatings, magnesium alloys, electrochemical impedance spectroscopy

## Abstract

Coating of an organo-silane (Bis-1,2-(TriethoxySilyl)Ethane (BTSE)) has been observed to improve the corrosion resistance of magnesium alloy AZ91D. Three different types of surface preparations have been employed before condensing the silane coating on to the substrate. Corrosion resistance was investigated using electrochemical impedance spectroscopy (EIS). A specific alkali treatment of the substrate prior to the coating has been found to improve the corrosion resistance of the coated alloy, which has been attributed to the ability of the treatment in facilitating the condensation of a relatively compact siloxane film.

## 1. Introduction

### 1.1. Corrosion of Magnesium Alloys

High strength-to-weight ratio of magnesium and its alloys have made them the widely investigated materials for applications where weight reduction is of utmost importance (e.g., automobiles, aircraft structures) [1], as well as the non-toxic nature of magnesium has also attracted attention for use of magnesium alloys in bioimplant applications [2,3,4,5]. However, corrosion resistance of magnesium alloys is unacceptably poor for applications where corrosion resistance may be a criterion. Standard reduction potential for pure magnesium is −2.37 V with respect to standard hydrogen electrode (SHE). This is the lowest for any metal/alloy used for structural applications. The high electronegativity of magnesium and its alloys greatly amplifies the importance of problem of corrosion as compared to that for other metals and alloys used in commercial applications [1,2,3,4].

Although several magnesium alloys have been developed over the last decades, AZ-series alloys are still the most commonly used. Poor corrosion resistance continues to be the major obstacle in wider use of magnesium alloys, including the AZ-series alloys. Therefore, various approaches have been explored for amelioration/mitigation of corrosion problem, such as surface pre-treatments and use of organo-silane coatings [6,7].

### 1.2. Organo-Silane Coatings for Improvement in Corrosion Resistance

Surface treatments based on chromate conversion coatings have been widely used for corrosion protection of metallic engineering materials including magnesium alloys. However, increasing concerns about carcinogenic nature of Cr(VI) and the pertaining regulatory restrictions have prompted rapid research into development of alternative surface treatments [8], such as other conversion coatings [9,10,11], graphene-based coatings [12], metallic coatings [13] and polymeric coatings [14]. Silane based coatings are among the environment friendly polymeric coatings. Though silane coatings possess excellent adhesion properties that enable their use as paint undercoats, even standalone silane films on metallic substrates confer considerable corrosion resistance [6,7]. Silane coatings have been successfully applied by sol-gel processing route for corrosion resistance of aluminium alloys, steels, magnesium alloys and other metallic substrates [7,15,16,17,18,19,20,21,22,23,24,25,26,27]. Silane has been investigated considerably for surface protection of magnesium alloys, as a component of composite coatings [28,29,30], and as a pretreatment for polymeric coatings [31]. For standalone silane coatings on magnesium alloys, pretreatments such as plasma electrolytic oxidation has been investigated for enhancing adhesion of silane with the alloy substrates [32,33,34]. In application of silanes as coatings on metallic surfaces, silane molecules form chemical bonds with the metal (M) substrate by condensation of silanol groups of hydrolysed silane molecules. Upon ageing and curing, cross-linked structures of M-O-Si and Si-O-Si bonds develop whose characteristics determine ability of the coating to provide corrosion resistance. Since the silanes bond with surface oxide/hydroxide layers upon condensation, substrate’s surface characteristics is expected to play a crucial role in the stability and adhesion of the silane films. A suitable alkaline pre-treatment can result in generation of sufficient hydroxyl groups at the substrate surface for facilitating homogeneously distributed condensation of silane [6,7]. For examples, condensation of silane films on alkali-pre-treated aluminium alloys and the surface coverage of the substrate by silane is reported to increase with the hydroxyl groups [27]. It is necessary to develop similar understanding for magnesium alloys. Accordingly, this study investigates role of a few hydroxide pre-treatments and subsequent silane coating on corrosion resistance of a magnesium alloy, AZ91D. Electrochemical impedance spectroscopy was employed for thorough characterisation of the corrosion resistance.

## 2. Materials and Methods

### 2.1. Test Materials

#### 2.1.1. Alloy Sample Preparation and Surface Treatments

Coupons (3 × 5 × 2 cm^3^) machined out of a sand-cast ingot of AZ91D were ground from 120 to 2500 grit on SiC paper, rinsed with deionized water, degreased with ethanol and dried with compressed air. The coupons with three different surface treatments as described below were used:

1. Substrate ground to 2500 grit finish, degreased with ethanol and dried with compressed air (henceforth, referred as uncoated substrate).
2. Immersion of ground substrates (2500 grit finish) in 3 M NaOH for 48 h (with a view to achieving a uniform distribution of hydroxide on the surface).
3. Potentiostatic polarization of ground substrate (2500 grit finish) in 3 M NaOH for 600 s at 100 mV (with respect to SCE) to form hydroxide on surface. This potential (100 mV_SCE_) was selected based on the potentiodynamic polarization of pure magnesium in 3 M NaOH solution [35].

#### 2.1.2. Hydrolysis of Silane

Bis-1,2-(Triethoxy-Silyl) Ethane (BTSE) silane was procured from Gelest Inc. (Morrisville, PA, USA). Hydrolysis of BTSE was carried out in a solution of water and methanol. Accordingly, 4:6:89.4:0.6 volume ratio of silane, water, methanol and glacial acetic acid was used for hydrolysis and subsequent condensation. Since the hydrolysis reaction occurs in multiple steps, and using methanol as a solvent retards the kinetics of forward reaction, 0.6 mL of acetic acid per 100 mL of solution was added to promote the hydrolysis. The presence of methanol may also prevent completion of hydrolysis, and hence, the unhydrolysed groups may still be in the cured film after condensation. The pH of the hydrolysed silane solution was determined to be 4.5. The hydrolysis of BTSE employed in the present study is consistent with that in earlier studies [18,20,23,25,36].

#### 2.1.3. Coating AZ91D with BTSE

The coupons of AZ91D with surface conditions as described in Section 2.1.1 were immersed in the hydrolysed silane for 5 min and drip-dried in air for 15 min before curing in the ambient atmosphere of an oven at 120 °C for 1 h to facilitate the cross-linking among the silane molecules of the coating. The coupons were found to develop a shiny and transparent coating homogeneously covering the entire surface. A scanning electron microscope (model JOEL JSM 840A, Tokyo, Japan) and the attached energy dispersive X-ray spectroscope (EDX) were used to qualitatively characterize the chemical homogeneity of the coatings.

### 2.2. Electrochemical Impedance Spectroscopy of Coatings

Electrochemical impedance spectroscopy (EIS) has been widely used for the investigation of such properties of coatings that are directly associated with the corrosion resistance, viz., delamination, electrolyte uptake and porosity [36,37,38,39]. For a given set of such EIS data, a given equivalent electrical circuit (EEC) would be selected that fit the set of data best. The components of the proposed EEC represent various layers and interfaces of the system, and the electrochemical response across such interfaces. Proper selection and interpretation of EEC is critical since an incorrect model can lead to misleading representation of corrosion or protection phenomenon/mechanism [37]. The pioneering studies on implementation of EIS for corrosion characterization e.g., Mansfeld, et al. [39] suggested the use of a modified Randles cell as EEC, from which a large number of other models can be derived. These models have been very popular; however, the physical interpretations of these models do not correspond to many real-life systems. Therefore, it became necessary to consider the relevant physical properties of the system while proposing an appropriate EEC. It has been a general practice to interpret the EIS data based on visible time constants and correlating them to capacitance and resistance in the employed models. However, with the advent of sophisticated softwares, it has been possible to rely on Bode and Cole-Cole plots for fitting and simulation, as these plots are more sensitive to small changes in capacitance and resistance in the circuits as compared to Nyquist plots that were extensively employed when EIS was first implemented for corrosion characterization [36,37,38,39]. Different types of EEC have been proposed to model coatings, oxide layers and electrical double layers including properties such as porosity and hydrophobicity of the coatings [38]. In the present work, EECs based upon hypothetical corrosion mechanism, taking the physical properties of coatings into consideration, was followed.

EIS tests were carried out using PAR Potentiostat/Galvanostat model 2273 and a typical three electrode corrosion cell containing 300 mL of the test solution. An area of 0.78 cm^2^ of the working electrode was exposed to the test solution while an electrical connection was made to the potentiostat from the opposite side of the working electrode. The electrochemical cell consisted of a saturated calomel electrode (SCE) used as the reference electrode and a platinum wire mesh used as the counter electrode. EIS tests were carried out in 0.1 M NaCl solution. A moderately corrosive test solution was chosen because a more benign solution would be too mild for meaningful corrosion tests whereas a more concentrated solution could have increased the scatter in the impedance spectrum, particularly in the case of a highly anodic material such as magnesium alloy. Open circuit potential (OCP) was monitored for a certain period of time (generally for 1 h) to ensure the stability of OCP with time. A fluctuation in OCP within 10 mV over a period of 1000 s was assumed as stable potential for carrying out electrochemical tests. EIS tests were carried out by applying a sinusoidal potential wave at OCP with an amplitude of 10 mV. Impedance response was measured over frequencies between 1 MHz and 10 mHz, recording 10 points per decade of frequency using Princeton Applied Research (PAR) PowerSuite Electrochemistry package. Impedance analysis was carried out using PAR ZSimpWin package for Windows for frequencies between 100 kHz and 30 mHz to prevent misinterpretation of any artefact that may be present in high frequency region, or the scatter in low frequency region.

Chloride ions have been known to degrade the corrosion resistance of the coatings over time [40,41,42,43]. Since sodium sulphate solutions are relatively benign, hence the surface layers developed in sodium sulphate are expected to be electrochemically stabler and hance, more suitable for EIS measurements. Critical tests in sodium sulphate solution can be used to validate the EEC results. Accordingly, comparison of EIS test results conducted in 0.01 M Na_2_SO_4_ solution to those conducted in 0.1 M NaCl solution has been used as verification and test the applicability of the EEC model.

EIS tests were at least duplicated under each condition to ensure the reproducibility of results.

## 3. Results and Discussion

### 3.1. Surface Morphology of the Coated Alloy

**Figure 1:**
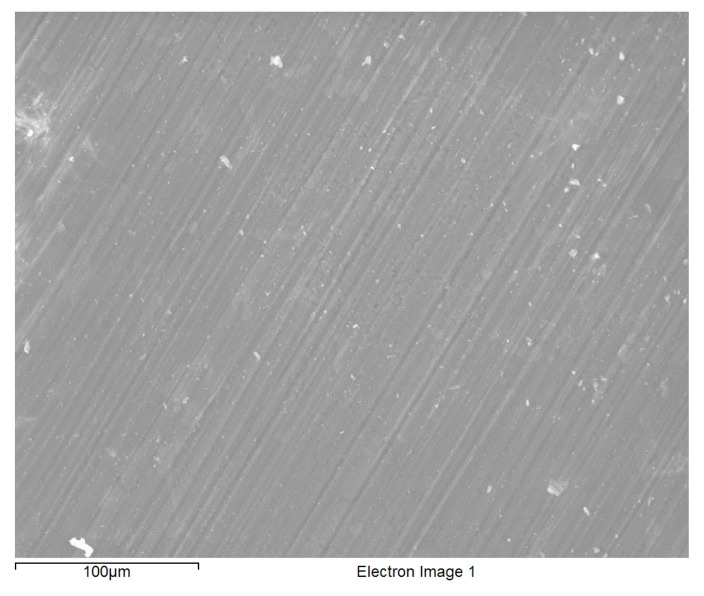
Typical BTSE coated AZ91D alloy surface.

**Figure 2:**
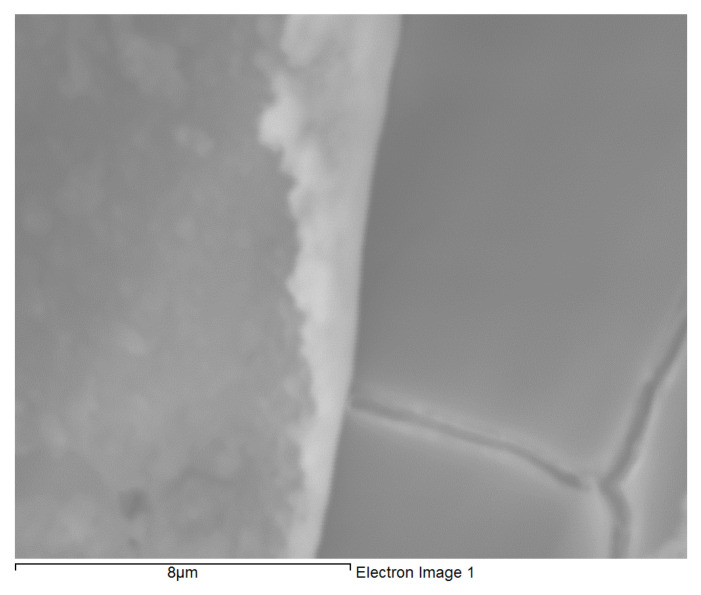
Delamination in BTSE coating on AZ91D exposed to 0.1 M NaCl for 100 h, that enabled determination of coating thickness.

Uniform, shiny and transparent coatings were formed upon condensation and subsequent curing, on each of the three types of substrates as described in the experimental details (Section 2.1.3). The coatings in each case was found to be completely covering the substrate with little signs of cracks or uncoated areas as suggested by the SEM micrograph of a typical BTSE coated AZ91D (Figure 1). EDXS spectra of freshly formed films on each of the coated substrates consisted the characteristic peaks of magnesium, aluminium and zinc (from the substrate), and silicon, carbon and oxygen from the silane film. EDXS analyses and SEM images inferred the substrates to be uniformly coated. However, the coatings occasionally suffered delamination during prolonged exposure to the corrosive NaCl solution (Figure 2); and the coating thickness from such delamination was determined to be about 500 nm.

### 3.2. Electrochemical Impedance Spectroscopy (EIS)

As elaborated in Section 2.2, EIS is a useful technique that has been widely employed for characterization of coated and uncoated metal surfaces and for understanding corrosion mechanisms. The technique is particularly sensitive to characterisation of pores and water uptake, and hence can detect deterioration of degrading coatings [16,37,39,42].

In the present study, the impedance data on the interfaces of substrate/surface oxide (hydroxide)/NaCl solution and substrate/surface oxide (hydroxide)/silane/NaCl solution were analysed using an equivalent electrical circuits (EEC). Various EEC’s that are reported in the literature were employed to simulate the data sets to corroborate the electrochemical impedance of the coated and uncoated substrates. However, the simulated data sets thus generated were found to be inconsistent with the observed impedance data. Hence, new EECs were developed for different interfacial scenarios based on the hypothetical corrosion mechanisms. The experimental data set, thus generated, was compared with the simulated data set to validate the hypothetical corrosion mechanisms for difference scenarios. The simulated impedance plots (Nyquist and Bode plots) were found to be in good agreement with the observed impedance data. It was possible to calculate the interfacial resistance and capacitance that can be related to homogeneity of the films and corrosion mechanisms. The relative magnitudes of these components provide an estimation of protection provided by coatings against the NaCl solution.

As-ground and air-dried surface of ZA91D alloy reacts with atmospheric oxygen and imminently forms a MgO layer on the surface. The atmospheric moisture can also react with the surface resulting in an occasional formation of a mix of oxide and hydroxide [43]. An immersion in NaOH solution as well as potentiostatic polarization in NaOH at passivating potentials develops a hydroxide layer on a magnesium alloy. Immersion of the alloy substrate in the silane coating bath and subsequent curing facilitates silane molecules’ bonding with the metal hydroxide developed upon prior immersion or polarization in NaOH [44]. When exposed to the chloride solution during corrosion test, the coating-substrate interface behaves as a capacitance with some inherent resistance to charge transfer processes, which formed the basis of electrical equivalent circuit models that was employed for developing the understanding of the mechanism of corrosion of silane coated and uncoated AZ91D.

**Figure 3:**
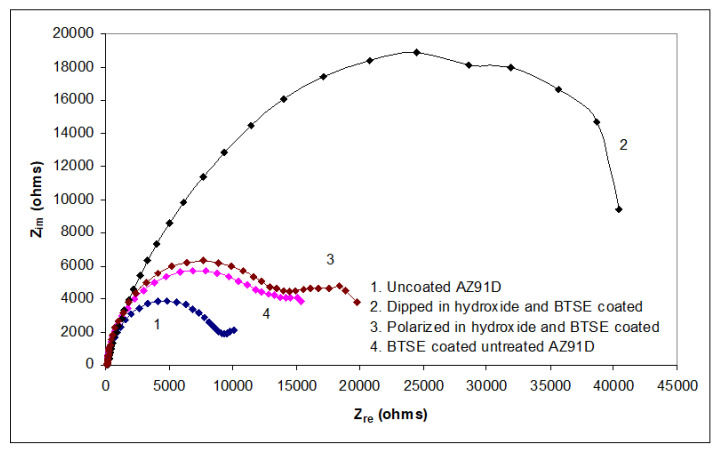
Nyquist plots for AZ91D in 0.1 M NaCl solution: (1) uncoated, (2) pre-dipped in hydroxide for 48 h and BTSE coated, (3) pre-polarized in hydroxide and BTSE coated, and (4) BTSE coated without any pre-treatment. Z_im_ and Z_re_ stand for imaginary and real impedances respectively.

EIS was carried out on the uncoated and BTSE-coated AZ91D with each of the three types of surface preparations described in Section 2.1. Nyquist plots are presented in Figure 3. The substrate with prior dipping in NaOH solution for 48 h followed by coating with BTSE provides considerably superior corrosion resistance as compared to the uncoated substrate as well as the substrates with other surface preparations followed by BTSE coating. Polarization resistance of the 48 h-dipped-BTSE-coated alloy is in excess of 40 kΩ whereas those of polarized (and coated) and untreated (and coated) is only 20 kΩ. Polarization resistance of uncoated substrate is close to 10 kΩ. A prior prolonged exposure to hydroxide would facilitate development of homogeneous and compact hydroxide layer that improves bonding of the silane film with the metallic substrate, thereby, improves the corrosion resistance of the coated substrate [15,16,17].

**Figure 4:**
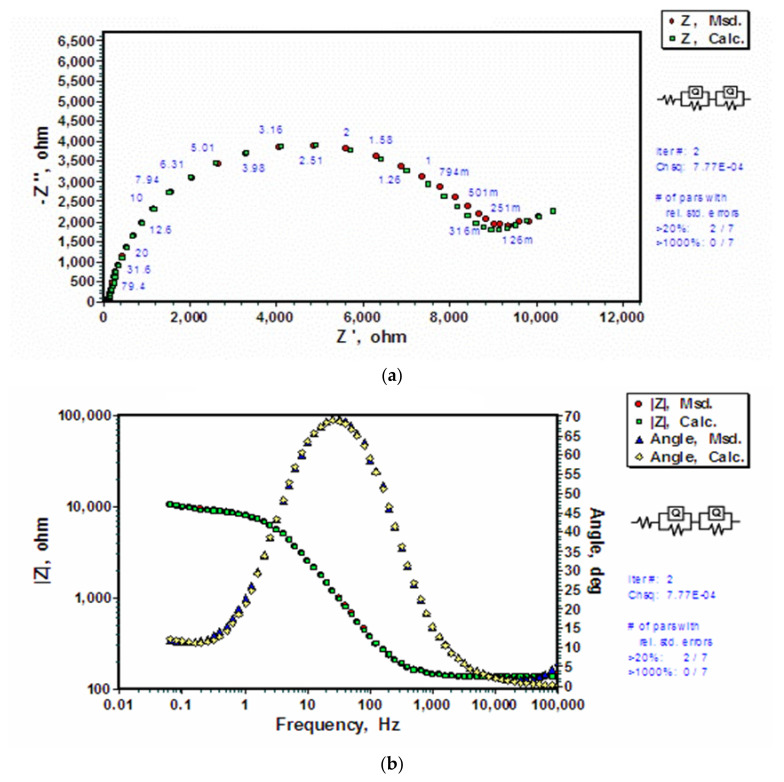
Experimentally measured and calculated data using the electrical equivalent circuit, R_s_(Q_o_R_o_)(Q_dl_R_dl_), for uncoated AZ91D alloy exposed to 0.1 M NaCl solution: (**a**) Nyquist plots for impedance data, and (**b**) Bode and phase angle plots (|Z| stands for impedance).

**Figure 5:**
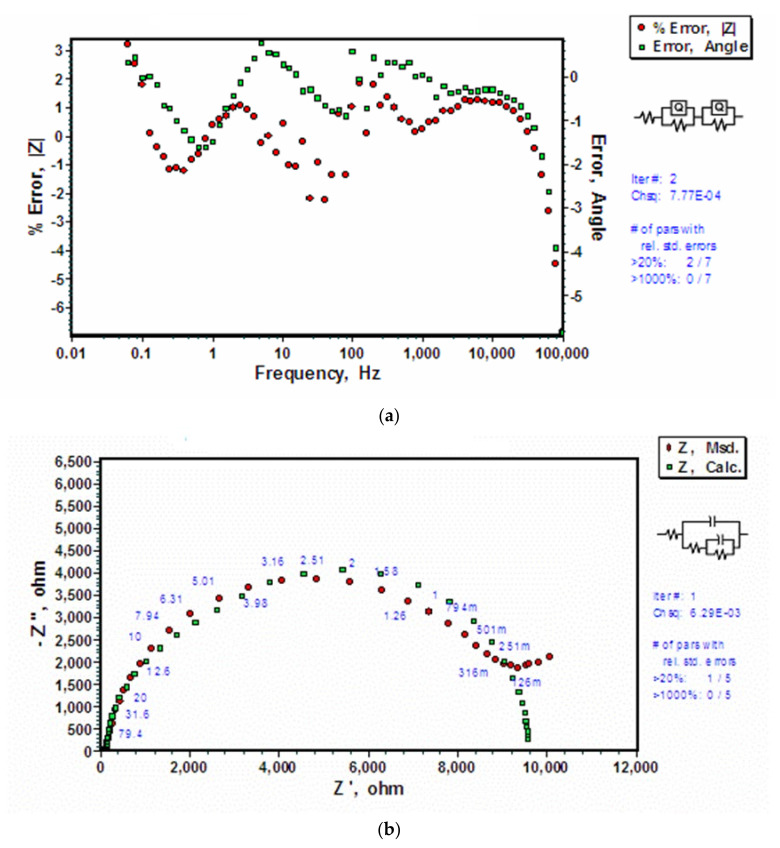
Validity checks for EIS data for uncoated AZ91D alloy exposed to 0.1 M NaCl solution: (**a**) Error plots for calculated values for |z| and angle for electrical equivalent circuit, R_s_(Q_o_R_o_)(Q_dl_R_dl_), and (**b**) Evidence of Nyquist plot not fitting well with another electrical equivalent circuit, (Rs(CoRo(C_dl_R_dl_))).

For an uncoated AZ91D, since there wes no silane coating, a simpler model could simulate the data corresponding to a hypothetical corrosion mechanism where the electrical double layer and the magnesium hydroxide layer each constitute a set of capacitance and resistance in parallel, and the two sets are placed in series (as shown in Figure 4). The hydroxide and double layer capacitance are expressed in terms of a constant phase element (CPE). It is the ‘power law dependent’ interfacial capacity which accounts for the topography of imperfections and roughness of substrate as well as the coating. A theoretical simulation was conducted using a R_s_(Q_o_R_o_)(Q_dl_R_dl_) electrical equivalent circuit (EEC) where solution resistance is represented by the first resistance R_s_, electrical double layer is represented by CPE (Q_dl_) and resistance (R_dl_) in parallel with surface oxide/hydroxide film interface acting as another series addition of CPE (Q_o_) and resistance (R_o_) in parallel. Estimation errors (Figure 5b), and a comparison with simulation results derived upon other models (e.g., one shown in Figure 5b) suggested R_s_(Q_o_R_o_)(Q_dl_R_dl_) model to be the closest fit to the impedance data with errors being less than 2.79%. The observed Nyquist and Bode impedance plots and simulation results for uncoated AZ91D alloy are shown in Figure 4. Nyquist plots show that the polarization resistance, as measured by the diameter of semi-circle, to be about 10 kΩ. Simulated and experimental spectra match very well both for the Nyquist and Bode plots (Figure 4a,b). Bode plot shows a time constant close to 100 Hz and another time constant at low frequencies (<1 Hz). The high frequency time constant is attributed to the frequency response of charge transfer controlled processes whereas the low frequency time constant accounts for the diffusion controlled mass transfer reactions [45]. Error plots (Figure 5a) show that the errors in simulated data are less than 3% for |z| and less than 5% for angle. Errors are generally random implying a close fit. Table 1 presents the theoretically calculated parameters for the applied EEC model, R_s_(Q_o_R_o_)(Q_dl_R_dl_).

In developing EEC, it is critical to understand that an incorrect hypothesis can lead to models and simulated impedance data that do not conform to the measured impedance data in the real systems. Figure 5b presents a typical example where a model (R_s_(C_o_R_o_(C_dl_R_dl_))), instead of R_s_(Q_o_R_o_)(Q_dl_R_dl_), was applied to generate data for simulating corrosion mechanism for uncoated AZ91D system. This model, that assumes partial coverage of exposed surface area and corrosion prevention by accumulation of corrosion products, does not account for the porosity of the corrosion product film. As a result, as is clearly seen, the simulated data are not in agreement with the observed impedance data (Figure 5b).

**Figure 6:**
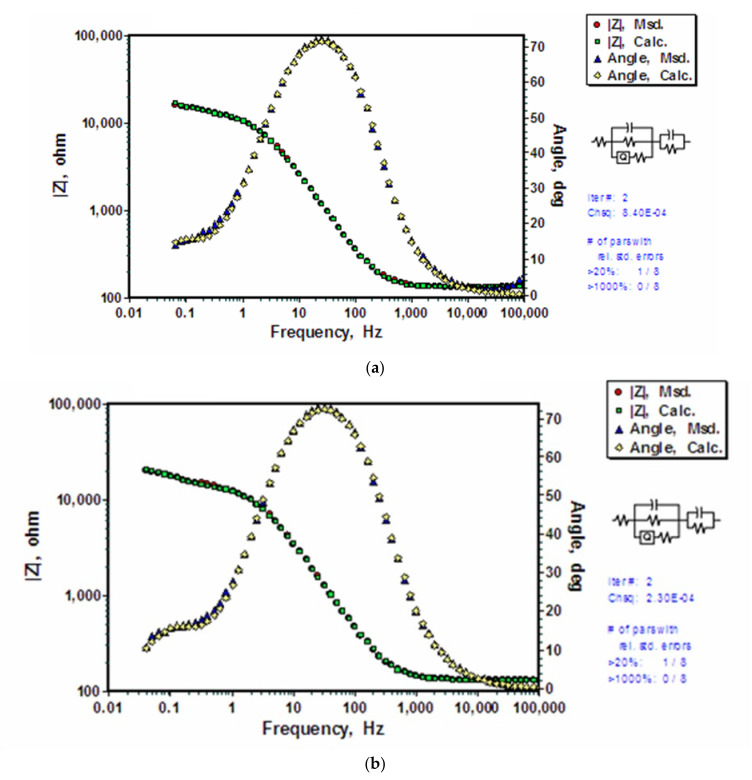
Experimentally measured Bode and phase angle plots and the corresponding data calculated using the electrical equivalent circuit, R_s_(C_Si_R_Si_(Q_h_R_h_))(C_dl_R_dl_) for coated AZ91D alloy exposed to 0.1 M NaCl solution: (**a**) BTSE coating on substrate with no prior alkali treatment, and (**b**) BTSE coating on substrate pre-polarized in hydroxide.

For BTSE coated substrates, EIS models were developed taking into consideration the additional layer, i.e., silane coating. A model that considers the silane film to be porous was found to be the best fit for the measured impedance data for the untreated-and-coated (Figure 6a) and pre-polarized-and-coated (Figure 6b) substrates. In the theoretical simulation using the EEC, R_s_(C_Si_R_Si_(Q_h_R_h_))(C_dl_R_dl_), solution resistance is represented by R_s_, silane coating is represented by the capacitance (C_Si_) and resistance (R_Si_) in parallel with the surface oxide/hydroxide film (Q_h_R_h_) acting as CPE. Interfacial electrical double layer is included as another series addition of a set of capacitance (C_dl_) and resistance (R_dl_) (which are themselves in parallel). The calculated parameters for the applied EEC, R_s_(C_Si_R_Si_(Q_h_R_h_))(C_dl_R_dl_) are presented in Table 1. It is noted that the impedance values of electrical double layer for uncoated AZ91D (Q_dl_ and R_dl_) are similar to those in silane coated AZ91D (C_dl_ and R_dl_) indicating similar charge transfer processes. It is also noted that the capacitance for silane coating and that of the oxide/hydroxide layer underneath the coatings are very similar (of the order of 1 µF). The capacitance (2.74 µF) of silane coating developed on the pre-polarized substrate with hydroxide is smaller than that of coating on untreated substrate (4.10 µF) indicating that there was less water uptake by BTSE coating when the substrate was pre-polarized. This is in agreement with the higher total polarization resistance of the pre-polarized and coated substrate as seen in Nyquist plots in Figure 3. The capacitance assigned to the electrical double layer (C_dl_) is of the order of 1 E2 µF which is similar to that for the double layer capacitance of uncoated AZ91D (Table 1).

The data generated using EEC, R_s_(C_Si_R_Si_(Q_h_R_h_))(C_dl_R_dl_) model match very well with the experimentally determined EIS data. The change in the parameters for silane film, hydroxide layer, electrical double layer and solution resistance is consistent for untreated-and-coated (Figure 6a) and pre-polarized-and-coated (Figure 6b) substrates. However, the physical interpretation of the model conflicts with structure of the silane film. The suggested model represents a silane coating in parallel with hydroxide layer, both of which need to be delaminated to a certain extent to allow the presence of electrolyte at the substrate interface, which is essential for explanation of the presence of double layer in series with the silane film as well as with the hydroxide layer. However, no such delamination was observed, and thus, R_s_(C_Si_R_Si_(Q_h_R_h_))(C_dl_R_dl_) model was deemed invalid for the untreated-and-coated and pre-polarized-and-coated substrates. The R_s_(C_Si_R_Si_(Q_h_R_h_))(C_dl_R_dl_) model was found to be invalid also for the substrate with BTSE coating upon prior dipping. The calculated parameters for individual components are listed in Table 2. It is clear that the simulated data do not fit well with the experimentally determined data for solution resistance and silane coating capacitance.

Since the experimental procedure was the same for the three sets of data shown in Figure 6, the discrepancy described in the preceding section can only be attributed to the use of an incorrect model for the coated substrates.

**Figure 7:**
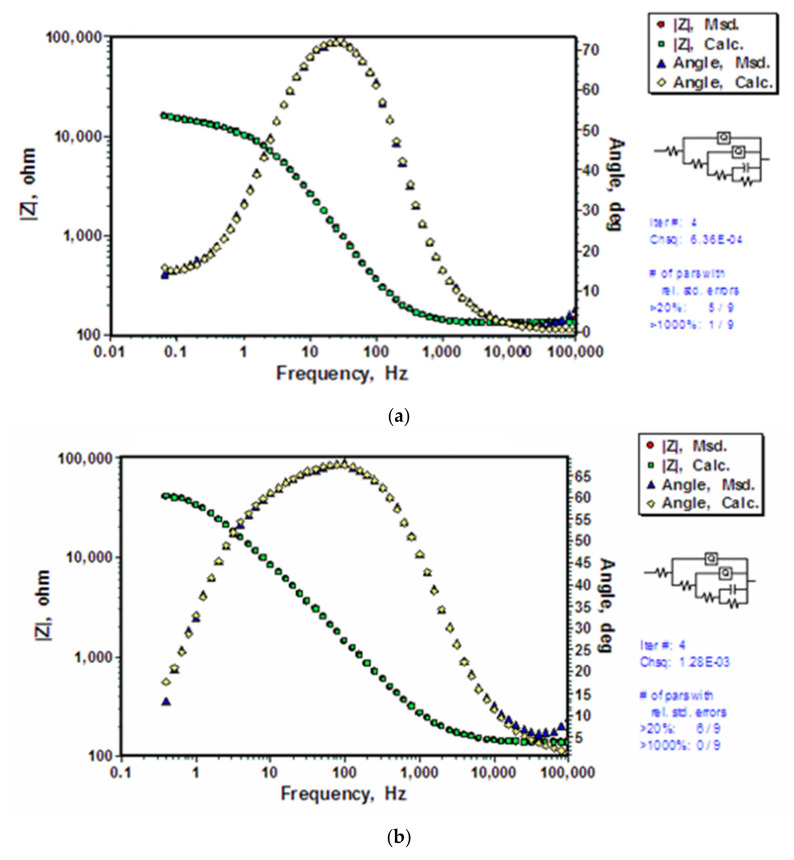

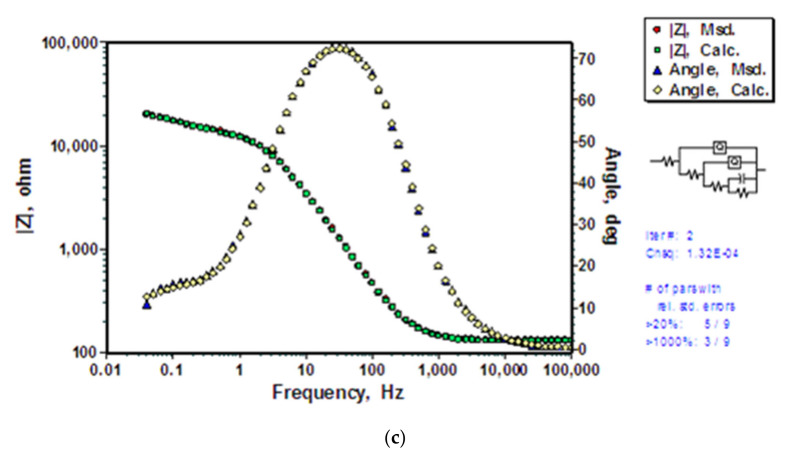
Experimentally measured Bode and phase angle plots and the corresponding data calculated using the electrical equivalent circuit, R_s_(Q_Si_(R_Si_(Q_h_(R_h_(C_dl_R_dl_))))) for coated AZ91D alloy exposed to 0.1 M NaCl solution: (**a**) BTSE coating on substrate with no prior alkali treatment, (**b**) BTSE coating on substrate pre-polarized in hydroxide, and (**c**) BTSE coating on substrate pre-dipped in hydroxide for 48 h.

The improved polarization resistance of the coated substrates (as seen in Figure 3) is attributed to the characteristics of the silane coating. This hypothesis should be able to be validated when a suitable/appropriate EEC is employed. An EEC, R_s_(Q_Si_(R_Si_(Q_h_(R_h_(C_dl_R_dl_))))), which considers nested QR circuit as electrical equivalent of silane coating with improved cross-linking, was employed to represent the untreated-and-coated, pre-polarized-in-alkali-and-coated and pre-dipped-in-alkali-and-coated substrates (Figure 7a–c). The simulation results were consistent with the unchangeable parameters observed in other coatings (R_s_ in Table 1 and Table 2), validating the hypothesis and the use of nested EEC. In the theoretical simulation using a R_s_(Q_Si_(R_Si_(Q_h_(R_h_(C_dl_R_dl_))))) EEC, the solution resistance is represented by R_s_, silane coating is represented by a CPE (Q_Si_) and resistance (R_Si_) in parallel with oxide/hydroxide layer acting as CPE (Q_h_(R_h_)). The inclusion of oxide/hydroxide film as a secondary loop corresponds to the improved coating characteristic (such as cross-linking), thus, the lesser porosity within the film. Electrical double layer was included as another loop addition consisting of capacitance (C_dl_) and resistance (R_dl_) in parallel. The nested loops are the mathematical representation of interconnected porosities which act as tortuous conducting path for electrolyte to reach the substrate, forming an electrical double layer at the interface. Since the EEC employed here considers improved coating characteristic, indicating that the underlying hydroxide film remains stable, and subdues the corrosion reactions even after one hour of immersion.

The calculated parameters for the applied EEC R_s_(Q_Si_(R_Si_(Q_h_(R_h_(C_dl_R_dl_))))) are presented in Table 3. The capacitance of the silane film (Q_Si_-Y_o_) is slightly lesser for the silane coating developed on the substrate pre-dipped in hydroxide as compared to the capacitance of coating developed on the untreated or pre-polarized substrates. The lower capacitance after immersion in electrolyte indicates lesser water uptake by the silane coating on the substrate pre-dipped in hydroxide. Resistance of the silane film (R_Si_) is also greater for the film developed on the substrate dipped in hydroxide as compared to other substrates. Lesser water uptake and improved resistance indicate improved coating characteristic of the silane film.

Capacitance is directly proportional to the area of capacitor and inversely proportional to the thickness between the plates of the capacitor. Data in Table 3 suggest the capacitance assigned to hydroxide layer (Q_h_-Y_o_) on the substrate dipped in hydroxide for 48 h to be two orders of magnitude smaller than that for the hydroxide layers on other substrates (i.e., those of untreated and polarized substrates), indicating either relatively smaller area or a more compact hydroxide coating that developed upon dipping in hydroxide for 48 h. Since the area of the ground substrate remains unchanged, the smaller capacitance can only be attributed to the development of a more compact film upon dipping in 3 M NaOH solution for 48 h. The coefficient, Q_h_-n being 1 indicates this coating to be a purely capacitive, which improved the overall corrosion resistance, as indicated in Figure 3. A relatively compact film and the improved number of hydroxide sites, that are presumed to have been achieved after dipping for 48 h in hydroxide produced a silane coating that possesses a lower capacitance of the silane film (Q_Si_-Y_o_ in Table 3) and improved polarization resistance in Nyquist plots (Figure 3).

The solution resistance, R_s_ is similar in all the four cases (Table 1, Table 2 and Table 3), indicating close similarities in solution and set-up impedance. Changes in the electrical double layer properties (C_dl_ and R_dl_) cannot be analysed using the results of this model. The errors appearing in the calculations for C_dl_ and R_dl_ were too high to reveal any meaningful information from comparisons.

**Figure 8:**
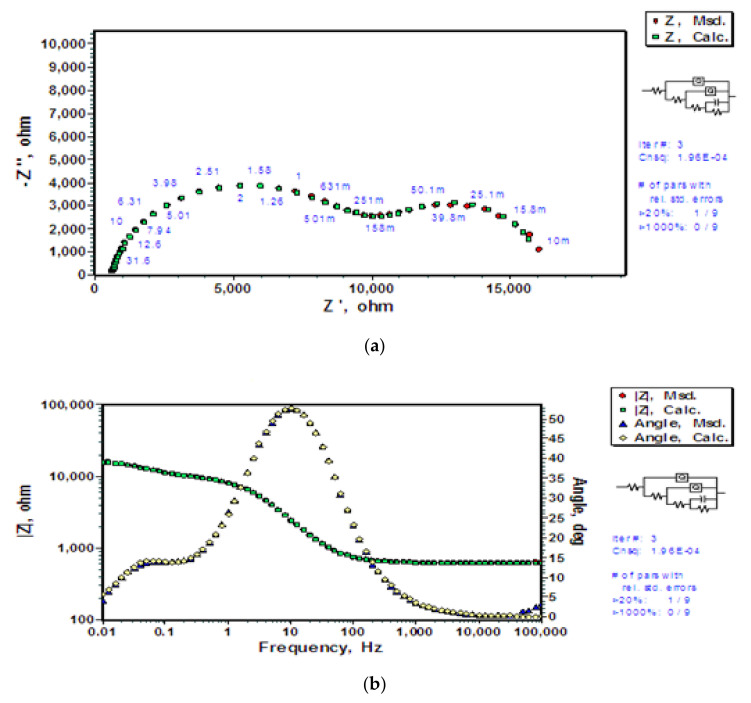
Experimentally measured and the corresponding data calculated using the electrical equivalent circuit, R_s_(Q_Si_(R_Si_(Q_h_(R_h_(C_dl_R_dl_))))) for coated AZ91D alloy pre-dipped in hydroxide for 48 h followed by BTSE coating and then exposed to Na_2_SO_4_ solution: (**a**) Nyquist plots, and (**b**) Bode and phase angle plots.

The observed impedance data from EIS tests and the simulated data using R_s_(Q_Si_(R_Si_(Q_h_(R_h_(C_dl_R_dl_))))) are in agreement for all the three types of substrates (the untreated-and-coated, the pre-polarized-in-alkali-and-coated, and the pre-dipped-in-alkali-and-coated), and the observed changes in the capacitive and resistive parameters can be well explained by the differences in pre-treatments and their effects on coatings. However, carrying out EIS experiments using a less corrosive electrolyte may be useful in validation of the applied EEC (R_s_(Q_Si_(R_Si_(Q_h_(R_h_(C_dl_R_dl_)))))) for verification the hypothetical corrosion mechanism on which the EEC is based. Thus, the EIS tests were carried out on BTSE coated AZ91D (with prior dipping for 48 h in hydroxide) in 0.01 M Na_2_SO_4_ solution, and R_s_(Q_Si_(R_Si_(Q_h_(R_h_(C_dl_R_dl_))))) was applied to simulate the corresponding data. The calculated parameters for the applied R_s_(Q_Si_(R_Si_(Q_h_(R_h_(C_dl_R_dl_))))) model are presented in Table 4. The observed and simulated EIS data (using R_s_(Q_Si_(R_Si_(Q_h_(R_h_(C_dl_R_dl_)))))) are presented in Figure 8a,b. The increase in electrolyte resistance (R_s_ = 622.6 Ω in Table 4 as compared to R_s_ = 137.5 Ω in Table 3) is responsible for the difference in the nature and shape of Nyquist and Bode plots. Two medium frequency capacitive loops, that merged in the Nyquist plot in 0.1 M NaCl (Figure 3), are distinctly visible in Nyquist plots in 0.01 M Na_2_SO_4_ solution (Figure 8a). The capacitance of the silane coating (Q_Si_-Y_o_) that is about an order of magnitude greater in 0.01 M Na_2_SO_4_ solution suggests a greater water uptake in the silane coating. Since the coating for the two tests were developed identically, the coating parameters (Q_Si_-Y_o_ and R_Si_) are comparable (Table 3 and Table 4). The fact that chloride ions react with magnesium hydroxide and dissolve the barrier layer is indicated by considerable difference in the capacitance of the hydroxide layer. The capacitance of the hydroxide layer in 0.1 M NaCl solution is two orders of magnitude lower than that in 0.01 M Na_2_SO_4_ solution, indicating a rapid decrease in the area covered by hydroxide upon exposure to the chloride-containing electrolyte. This would only be possible by the dissolution of the hydroxide layer present on the substrate and exposing larger area to corrosion due to chloride ions. Low conductivity of the electrolyte also results in a greater thickness of the electrical double layer. Since the capacitance of the electrical double layer is inversely proportional to the thickness, the higher double layer capacitance (C_dl_) in 0.01 M Na_2_SO_4_ solution is consistent with the ongoing argument.

In the above discussion, different EECs have been applied to describe the hypothetical corrosion mechanisms and behaviour of the coatings and various interfaces. The simulated data for R_s_(Q_Si_(R_Si_(Q_h_(R_h_(C_dl_R_dl_))))) is consistent with the observed impedance data, thus establishing the validity of the EEC in describing the corrosion models of uncoated and BTSE coated AZ91D (with two types of alkali pre-treatments). The EIS results and parameters based on the EEC, R_s_(Q_Si_(R_Si_(Q_h_(R_h_(C_dl_R_dl_))))), can explain the corrosion behaviour of coated substrates using different electrolytes. As further evidence, the change in electrolyte changes the behaviour of Nyquist and Bode plots; however, the changes are consistent with the hypothesized mechanism. Thus R_s_(Q_Si_(R_Si_(Q_h_(R_h_(C_dl_R_dl_))))) model indeed represents the coating and degradation of the coating upon exposure to chloride.

## 4. Conclusions

Role of specific alkaline surface pre-treatments on the performance of BTSE coating on magnesium alloy AZ91D has been investigated. EIS was used to study BTSE coatings on three different types of surface preparation involving induced hydroxide (prior dipping in NaOH and polarizing at passivating potential in NaOH). EIS models were developed for the hypothetical corrosion mechanism in each case. The corrosion performance of coatings with prior alkali treatments was compared to that of coating on untreated AZ91D. The results indicate that BTSE coatings improve the corrosion resistance of the coated substrates, as evidenced by the EIS results. Coating developed on the alloy surface prepared by dipping in NaOH solution was the most propitious for corrosion resistance. Prior-dipping in hydroxide would result in a relatively homogeneous and compact hydroxide layer that improves charateristics of silane film for corrosion resistance.

## Figures and Tables

**Table 1:** Corrosion of uncoated AZ91D: Calculated parameters for components in Model R_s_(Q_o_R_o_)(Q_dl_R_dl_), after immersion in 0.1 M NaCl solution.

Parameter	Estimated Value	Standard Error (%) ^#^
R_s_ (Ω)	138.1	0.6719
Q_o_-Y_o_ (F)	8.19 × 10^−6^	2.543
Q_o_-n	0.9226	1.145
R_o_ (Ω)	8463	4.138
Q_dl_-Y_o_ (F)	0.000595	19.9
Q_dl_-n	0.7571	20.78
R_dl_ (Ω)	7288	53.67

^#^ Errors in impedance data ≤ 2.79% and χ^2^ = 7.769 × 10^−4^. R_s_: solution resistance; Q_o_ and R_o_: CPE and resistance of surface oxide/hydroxide film; Q_dl_ and R_dl_: CPE and resistance of electrical double layer; Y_o_: admittance of respective CPE; n: exponent of the angular frequency of respective CPE.

**Table 2:** Corrosion of BTSE coated AZ91D, untreated, polarized in hydroxide and dipped in hydroxide, after immersion in 0.1 M NaCl solution: Calculated parameters for components in Model R_s_(C_Si_R_Si_(Q_h_R_h_))(C_dl_R_dl_).

Parameter	Untreated Substrate		Substrate Polarized in Hydroxide		Substrate Dipped in Hydroxide	
	Estimated Value	Standard Error (%)	Estimated Value	Standard Error (%)	Estimated Value	Standard Error (%)
R_s_ (Ω)	134.7	0.74	131.6	0.41	3.32 × 10^−4^	5.82 × 10^7^
C_Si_ (F)	4.10 × 10^−6^	18.41	2.74 × 10^−6^	8.94	1.29 × 10^−9^	279.6
R_Si_ (Ω)	1.37 × 10^4^	3.05	1.43 × 10^4^	1.01	9.35 × 10^3^	8.36
Q_h_-Y_0_ (F)	7.20 × 10^6^	8.05	4.54 × 10^−6^	3.69	4.99 × 10^−6^	4.40
Q_h_-n	0.70	10.67	0.78	3.40	0.81	0.51
R_h_ (Ω)	145.7	443.9	103	101	139.5	141.7
C_dl_ (F)	4.28 × 10^−4^	13.51	2.72 × 10^−4^	4.61	3.83 × 10^−6^	4.06
R_dl_ (Ω)	6.83 × 10^3^	11.64	7.91 × 10^3^	2.85	3.46 × 10^4^	1.98
Impedance data	2.90%		1.52%		2.23%	
Impedance data	8.40 × 10^−4^		2.31 × 10^−4^		4.97 × 10^−4^	

R_s_: solution resistance; C_Si_ and R_Si_: capacitance and pore resistance of silane coating; Q_h_ and R_h_: CPE and resistance of oxide/hydroxide film; C_dl_ and R_dl_: capacitance and resistance of electrical double layer; Y_o_: admittance of respective CPE; n: exponent of the angular frequency of respective CPE.

**Table 3:** Corrosion of BTSE coated AZ91D, untreated, polarized in hydroxide and dipped in hydroxide, after immersion in 0.1 M NaCl solution: Calculated parameters for components in Model R_s_(Q_Si_(R_Si_(Q_h_(R_h_(C_dl_R_dl_))))).

Parameter	Untreated Substrate		Substrate Polarized in Hydroxide		Substrate Dipped in Hydroxide	
	Estimated Value	Rel. Std. Error (%)	Estimated Value	Rel. Std. Error (%)	Estimated value	Rel. Std. Error (%)
R_s_ (Ω)	133.9	0.54	131.3	0.25	137.5	1.05
Q_Si_-Y_o_ (F)	7.58 × 10^−6^	3.62	5.83 × 10^−6^	1.40	2.87 × 10^−6^	8.54
Q_Si_-n	0.93	0.59	0.93	0.23	0.85	1.2
R_Si_ (Ω)	1.08 × 10^4^	14.08	1.33 × 10^4^	5.53	1.97 × 10^4^	40
Q_h_-Y_o_ (F)	1.4 × 10^−4^	30.22	1.82 × 10^−4^	50.75	9.01 × 10^−7^	145.8
Q_h_-n	0.529	64.37	0.68	30.14	1	35.87
R_h_ (Ω)	1.17 × 10^4^	128.5	26.67	2.14 × 10^11^	1.61 × 10^4^	75.26
C_dl_ (F)	5.02 × 10^−4^	90.52	7.75 × 10^−12^	2.05 × 10^9^	2.93 × 10^−6^	161.9
R_dl_ (Ω)	1.63 × 10^12^	2.36 × 10^10^	1.30 × 10^4^	4.39 × 10^8^	1.13 × 10^4^	112.7
Impedance data	2.52%		1.15%		3.58%	
Impedance data	6.37 × 10^−4^		1.32 × 10^−4^		1.28 × 10^−3^	

R_s_: solution resistance; Q_Si_ and R_Si_: CPE and pore resistance of silane coating; Q_h_ and R_h_: CPE and resistance corresponding to oxide/hydroxide film; C_dl_ and R_dl_: capacitance and resistance of electrical double layer; Y_o_: admittance of respective CPE; n: exponent of the angular frequency of respective CPE.

**Table 4:** Corrosion of BTSE coated AZ91D (with prior dipping in hydroxide), after immersion in 0.01 M Na_2_SO_4_ solution: Calculated parameters for components in Model R_s_(Q_Si_(R_Si_(Q_h_(R_h_(C_dl_R_dl_))))).

Parameter	Estimated Value	Standard Error (%)
R_s_ (Ω)	622.6	0.27
Q_Si_-Y_o_ (F)	1.13 × 10^−5^	1.88
Q_Si_-n	0.88	0.43
R_Si_ (Ω)	9.26 × 10^3^	1.68
Q_h_-Y_o_ (F)	2.90 × 10^−4^	17.08
Q_h_-n	0.98	11.04
R_h_ (Ω)	2.38 × 10^3^	22.47
C_dl_ (F)	7.47 × 10^−4^	19.23
R_dl_ (Ω)	3.87 × 10^3^	10.84
Impedance Data	1.40%	
Impedance Data	1.96 × 10^−4^	

R_s_: solution resistance; Q_Si_ and R_Si_: CPE and pore resistance of silane coating; Q_h_ and R_h_: CPE and resistance corresponding to oxide/hydroxide film; C_dl_ and R_dl_: capacitance and resistance of electrical double layer; Y_o_: admittance of respective CPE; n: exponent of the angular frequency of respective CPE.

## Data Availability

Data is contained within the article.

## Sample Availability

Samples of the materials used in this investigation are available with the corresponding author at Monash University.
